# Bilateral Papillophlebitis in a Patient with Mutation of Metilenetetrahydrofolate Reductase Enzyme

**DOI:** 10.4274/tjo.77785

**Published:** 2016-08-15

**Authors:** Hüseyin Güzel, Banu Turgut Öztürk, Şansal Gedik, Berker Bakbak, Abdullah Beyoğlu, Nadir Koçak

**Affiliations:** 1 Tatvan State Hospital, Ophthalmology Clinic, Bitlis, Turkey; 2 Selçuk University Faculty of Medicine, Department of Ophthalmology, Konya, Turkey; 3 Selçuk University Faculty of Medicine, Department of Medical Genetics, Konya, Turkey

**Keywords:** Papillophlebitis, hyperhomocycteinemia, methylenetetrahyrofolate reductase mutation

## Abstract

Papillophlebitis is known as central retinal vein occlusion seen in young patients. It usually presents as unilateral optic disc edema with cotton wool spots and hemorrhage in the peripapillary region. As it may be due to many autoimmune and inflammatory causes, a thorough systemic evaluation of the patient is warranted. In this case report we describe a bilateral, simultaneous papillophlebitis case thought to be related to hyperhomocysteinemia secondary to C677T polymorphism of methylenetetrahyrofolate reductase enzyme.

## INTRODUCTION

Papillophlebitis, considered a subtype of central retinal vein occlusion (CRVO), is a clinical condition more common in young adults. It usually presents unilaterally and localized to the area surrounding the optic disc. Fundus signs include disc edema and varying degrees of cotton wool-like peripapillary exudate and hemorrhage. Because papillophlebitis does not extend to the macula, visual acuity is mildly affected, but a wider blind spot is common on visual field analysis.^1,2^

Unlike CRVO, papillophlebitis spontaneously resolves, and its etiology also differs from that of CRVO. Whereas CRVO is generally associated with thrombus in older patients, the etiopathogenesis of papillophlebitis involves inflammation secondary to venous congestion.^1^ Therefore, it is advised to screen these patients for systemic diseases like hypertension, hyperlipidemia, aterosclerosis and diabetes mellitus as well as autoimmune and inflammatory diseases and thrombophilic risk factors such as Factor V Leiden mutation, vitamin B_6_ deficiency, folic acid deficiency, hyperhomocysteinemia, and Protein C and S deficiency.^1,2^

In this case report we describe a bilateral, simultaneous papillophlebitis case thought to be related to hyperhomocysteinemia secondary to C677T polymorphism of the methylenetetrahyrofolate reductase (MTHFR) enzyme.

## CASE REPORT

A 48-year-old male patient was referred from an eye clinic after presenting with complaints of transient loss of vision in both eyes. It was learned that the patient’s symptoms started in both eyes at the same time, 3 weeks earlier. The patient had a 1-year history of hypertension and was being treated with an angiotensin II receptor antagonist and hydrochlorothiazide combination (valsartan/hydrochlorothiazide) plus an adrenoreceptor antagonist (doxazosin mesylate).

**Figure 1:**
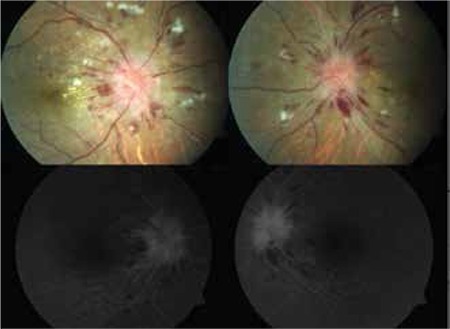
Fundus photography and fundus fluorescein angiography images of the patient at time of presentation

**Figure 2:**
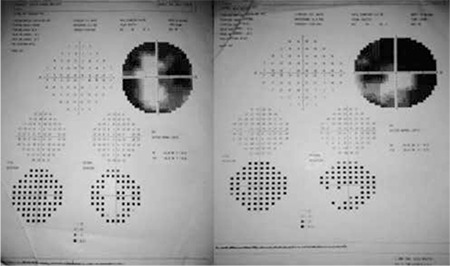
Right and left visual fields at presentation

On ophthalmologic examination, his BCVA was 0.6 (refraction values were cylinder -0.75, axis 180 D) in the right eye and 0.7 (sphere +0.50, cylinder -1.00, axis 175 D) in the left eye. His color vision evaluated by Ishihara test was 6/12 in the right and 10/12 in the left eye. Intraocular pressure measured by applanation was 14 and 16 mmHg in the right and left eyes, respectively. Anterior segment structures appeared normal and no cells were observed on slit-lamp examination. On fundus examination the optic discs of both eyes showed edema, indistinct margins and splinter hemorrhages extending from the disc to the periphery; intraretinal hemorrhage and cotton-wool exudates were also observed. No vitreous cells were detected. There were no signs of retinopathy like hypertensive arterial attenuation or artery-vein compression. On fundus fluorescein angiography, early hyperfluorescence at the optic disc and surrounding fields of hypofluorescence corresponding to areas of intraretinal hemorrhage were observed, but leakage consistent with vasculitis was not detected (Figure 1). Central macular thickness on optic coherence tomography (OCT) was 260 µm in the right eye and 237 µm in the left. His visual field (Humphrey 30-2) showed larger blind spots in both eyes; superior altitudinal scotoma and inferior arcuate scotoma were detected in the left eye, while concentric scotoma was present in his right eye (Figure 2).

The patient’s clinical findings were consistent with papillophlebitis, and visual evoked potential analysis revealed bilateral extended latencies. The patient was evaluated for etiologic factors. His arterial blood pressure was 180/90 mmHg and the Cardiology department was consulted for additional treatment. The patient had no history of an injury in his mouth, and pathergy test was negative. Sedimentation rate, level of C-reactive protein, and levels of protein C and protein S were normal. There were no signs on hepatic imaging suggesting tuberculosis or sarcoidosis. Cranial magnetic resonance imaging revealed bilateral gliotic changes in the temporal and parietal regions. In terms of thrombophilic risk factors, a homozygous (T/T) mutation of the MTHFR (C677T) gene was detected in the patient’s genetic analysis. The A1298C locus of MTHFR was normal. The patient had no family history of consanguineous marriage. Although his folic acid and vitamin B_12_ levels were within normal limits, his homocysteine level was measured as 32.63 µmol/L, twice the upper limit of the reference range (5.5-15 µmol/L). The hematology and cardiology departments were consulted and treatment for hyperhomocysteinemia was started.

**Figure 3:**
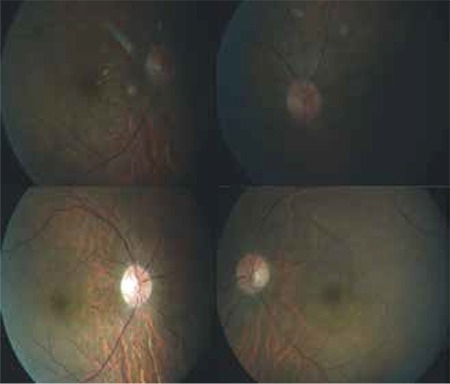
At 3 month follow-up (upper row), regression of the hemorrhage and sporadic exudate were observed; at 1 year follow-up (lower row), the exudates and hemorrhage were completely resolved

Over a 12-month follow-up period, the patient’s visual acuity reached 1.0 in both eyes. No neovascular signs were observed in the iris or angle on anterior segment slit-lamp examination. On fundus examination, regression of the optic disc edema and complete resolution of the intraretinal hemorrhage and cotton wool exudates were observed (Figure 3). Central macular thickness was 179 µm in the right eye and 180 µm in the left eye on OCT at 1 year.

## DISCUSSION

Papillophlebitis has also been referred to in the literature as benign retinal vasculitis, optic disc vasculitis, peripapillary retinal vein occlusion, blind spot enlargement syndrome or venous papillopathy. In addition to the lack of consensus regarding the name of this disease, its pathogenesis is also not fully understood.^2^ It has been proposed that papillophlebitis arises as a result of inflammation in the retinal veins near the optic nerve head which leads to CRVO. Others believe it may be a nonspecific inflammatory process secondary to certain hematologic or rheumatologic diseases. However, it has not been clearly determined what precipitates the condition. Hayreh3 described the disease as a unilateral vague haziness of vision and divided cases into two groups. The first group consisted of patients 30-35 years old with edema limited to the optic disc that resolved completely with nosequelae; the second group consisted of patients 26-55 years old with more severe peripapillary hemorrhage and CRVO involving the macula in 75% of cases.

Papillophlebitis is not generally associated with any comorbidities, though hypertension has been detected in 23-42% of patients and diabetes mellitus in 3-9%. Comparisons with other individuals in the same age groups revealed no differences in terms hyperlipidemia, hyperviscosity and hypercoagulability.^3^ The presence of hypertension in our patient suggests a predisposition to papillophlebitis. However, we also investigated serologic, biochemical, genetic and thrombophilic factors associated with other conditions possibly underlying papillophlebitis. In addition, because the patient exhibited bilateral optic disc edema, magnetic resonance imaging was done to exclude intracranial causes of optic disc edema. Biochemical analyses performed on our patient revealed hyperhomocysteinemia, and genetic analysis demonstrated a homozygous (T/T) mutation of MTHFR (C677T).

Homocysteine is a sulfur-containing amino acid created during methionine metabolism. Methionine is an essential amino acid which must be obtained through the diet. Homocysteine is metabolized in the body by either the transsulfuration or remethylation pathways.^4,5^ Vitamin B6 acts as a cofactor in the transsulfuration pathway, while folate and vitamin B_12_ are cofactors in the remethylation period. Deficiencies in these vitamins are associated with increased homocysteine levels. Because estrogen lowers plasma homocysteine levels, women have lower levels than men. Normal plasma homocysteine level is 5-15 µmol/L, and a value of 16 µmol/L or higher is considered hyperhomocysteinemia.^6^ Hyperhomocysteinemia is a major risk factor for paralysis,^7^ and is also a risk factor in arterial or venous thrombosis, myocardial infarction and chronic kidney failure.

Homocystinuria, an accepted variant of hyperhomocysteinemia, has been associated with lens dislocation, mental retardation and thromboembolic conditions. MTHFR deficiency is one of the common causes of hyperhomocysteinemia. Homozygous mutations in MTHFR lead to moderate to severe hyperhomocysteinemia and can cause early death. The most common MTHFR mutation is the C-T replacement in codon 677. The frequency of homozygotes for this polymorphism varies between 5-10% depending on the population. Homocysteine levels in these patients are in the 20-40 µmol/L range.^8^ Turaka et al.^9^ reported the case of a 15-year-old girl with unilateral papillophlebitis who had hyperhomocysteinemia and homozygous mutations in MTHFR C677T and A1298C. Smoking has also been determined as a risk factor for hyperhomocysteinemia. Our patient had a smoking history of 30 pack/years.

Although papillophlebitis resolves spontaneously, there have been attempts to speed recovery using different treatment protocols. Steroid therapy and anticoagulant therapy are two treatment protocols that have been administered to patients. There are proponents of intensive steroid therapy, while others cite that this may mask serious underlying conditions. Hayreh^3^ recommended systemic steroid therapy. He did not observe any beneficial effect of anticoagulant therapy. In the present case, the patient was followed without any steroid or anticoagulant treatment and his visual acuity returned to normal.

## CONCLUSION

Although visual prognosis is good, papillophlebitis may arise as the result of serious underlying systemic disease; therefore, performing the necessary genetic analyses to detect predisposing systemic, biochemical or thrombophilic etiologic factors is crucial for papillophlebitis patients. Hyperhomocysteinemia secondary to MTHFR mutation should be kept in mind as one of the possible causes of papillophlebitis.

### Ethics

Informed Consent: It was taken.

Peer-review: Externally peer-reviewed.
